# Excision of the Knee for Chronic Disease of the Joint

**Published:** 1875-08

**Authors:** 


### Excision of the Knee for Chronic Disease of the Joint—The Antiseptic Method.

At Guy's Hospital, London.—Service of Mr. Howse.

This woman, aged 27, has at various periods during the last eighteen years suffered with attacks of inflammation of the right knee-joint, which she states began subsequent to variola. Five years since, the inflammation was more severe than previously, and left the joint stiff; but she continued to walk upon the limb until five months ago, when it became so painful that she was unable to move it. The least movement of the limb causes acute pain. There is a certain amount of swelling of the joint, and there is evidently softening of the crucial ligaments, for the tibia and fibula are luxated backward and rotated somewhat outward. If the limbs are placed parallel, the eversion of the foot on the right side is easily seen, and the presence of disease is shown by rubbing the bones forcibly together. The articular cartilages are probably eroded, though there may be no bone absolutely denuded of its covering.

As in a case of this kind there is no use in attempting to cure the disease, and as extreme measures must be adopted, excision of the knee-joint shall be performed, and it shall be done by the antiseptic method. The limb is elevated, and the blood rubbed out towards the trunk with the hands; then the elastic bandage is applied, not all the way from the foot, however, but only around the thigh. The next step is to anoint the integument with carbolized oil, after which a circular incision is made below the patella, with a knife previously dipped in the antiseptic solution. This cut opens the joint, and is followed by a gush of pus and blood.

During all this time the assistants keep up a continuous spray of carbolized water upon the parts, using large atomizers containing a watery solution ( 1 to 40 ) of carbolic acid, in order to destroy all germs that may be in the air or upon the hands of the operator.

After the joint has been entered, the flaps are dissected up and the patella removed, when the bones are found covered with granulations and the cartilage is readily peeled off. A saw, with the blade set at an angle, is applied to the posterior surface of the tibia, and a section of the head removed, in one portion of which is found a loose sequestrum. In the head of the tibia there are seen two points of caseous bone, one on each side, which are removed by applying the saw transversely and cutting a triangular groove.

The soft tissues around the end of the femur are next divided, and the condyles sawn off; a caseous point similar to those in the head of the tibia being found, it is removed by the gouge and saw. The presence of this disorganized bone proves the propriety of the operation in this case, which in past times would have been left without operative interference and merely placed upon a splint.

Ligatures are now applied to the bleeding arteries by passing a needle, threaded with carbolized gut, through the tissues behind the vessels, tying securely, cutting off the ends, and leaving the knot in the wound. The parts come together nicely after the excision, so as to make a straight limb, and are kept in apposition by gut sutures. Finally, the parts are covered with the dry antiseptic gauze recommended by Mr. Lister, of Edinburgh, and a roller applied to the whole length of the limb.

The removal of this diseased joint will render the patient much more comfortable, and, if all goes well, will give her a limb which will enable her to walk without any difficulty.—*Phil. Med. Times.*

